# Supplementary material for: A high-resolution mRNA expression time course of embryonic development in zebrafish
Source: eLife. 2017 Nov 16;6:e30860. doi: 10.7554/eLife.30860 (PMC5690287; doi:10.7554/eLife.30860)
Supplement: Supplementary file 6. [file elife-30860-supp6.zip › biolayout-clusters-files/Cluster153-genes.html]

Cluster153


# Cluster153: Genes

| Ensembl ID | Gene Name | Chr | Start | End | Biotype |
| --- | --- | --- | --- | --- | --- |
| ENSDARG00000010149 | atp5a1 | 21 | 4916224 | 4927974 | protein\_coding |
| ENSDARG00000103364 | cs | 23 | 33981368 | 34001785 | protein\_coding |
| ENSDARG00000008785 | dldh | 25 | 26434503 | 26449804 | protein\_coding |
| ENSDARG00000007264 | gbas | 15 | 494880 | 504604 | protein\_coding |
| ENSDARG00000030765 | hadh | 1 | 49265846 | 49275216 | protein\_coding |
| ENSDARG00000003517 | impa1 | 2 | 27027279 | 27033274 | protein\_coding |
| ENSDARG00000030786 | mpi | 25 | 2909571 | 2925890 | protein\_coding |
| ENSDARG00000012387 | pdha1a | 5 | 23527514 | 23540924 | protein\_coding |
| ENSDARG00000005560 | ywhah | 10 | 17208071 | 17212122 | protein\_coding |
